# Dietary Factors and Higher Blood Pressure in African-Americans

**DOI:** 10.1007/s11906-014-0517-x

**Published:** 2015-02-04

**Authors:** Queenie Chan, Jeremiah Stamler, Paul Elliott

**Affiliations:** 1 Department of Epidemiology and Biostatistics, School of Public Health, Imperial College London, Norfolk Place, London, W2 1PG UK; 2 MRC-PHE Centre for Environment and Health, School of Public Health, Imperial College London, London, UK; 3 Department of Preventive Medicine, Feinberg School of Medicine, Northwestern University, 680 N Lake Shore Dr, Suite 1400, Chicago, IL 60611 USA

**Keywords:** African-Americans, Blacks, Blood pressure, DASH, Diet, Hypertension

## Abstract

Adverse blood pressure (BP) is a major independent risk factor for epidemic cardiovascular diseases affecting almost one third of the US adult population. This review synthesizes results from studies published over the past few years on BP differences and prevalent hypertension between US blacks and whites and their different intakes of foods (e.g., fruits, vegetables, and dairy products) and micronutrients (e.g., vitamin D, calcium, potassium, and phosphorus). Studies have consistently reported higher prevalence of adverse BP levels and hypertension and less favorable dietary intakes in blacks than in whites, but the influence of specific dietary factors on high BP risk for blacks remains unclear.

## Introduction

Adverse blood pressure (BP), prehypertensive and hypertensive, is an established major independent risk factor for epidemic cardiovascular diseases (CVD), afflicting a high proportion of the adult population worldwide [1]. In 2011–2012, approximately 29 % of US adults had hypertension (HTN) [2], defined as systolic blood pressure (SBP) 140 mmHg or higher and/or diastolic blood pressure (DBP) 90 mmHg or higher and/or the current use of antihypertensive medication [3]. Studies have consistently reported higher prevalence of adverse BP levels in blacks than in whites [2, 4–7]. Latest reports show that the prevalence of HTN in US adults was about 42 % for blacks and 28 % for whites [2]. Compared with whites, blacks develop high BP earlier in life, and their average BPs are higher [5, 8]. The higher BP levels for blacks are associated with the increased risk of heart disease and stroke [5, 9, 10]. Among adults with HTN, blacks had significantly (*P* < 0.05) higher mortality rates than whites for diseases of the circulatory system in the cohorts of the National Health and Nutrition Examination Survey (NHANES) [11]. Although the underlying explanations for these ethnic disparities remain poorly understood, they have been related to differences in the environment and lifestyles, such as education and socioeconomic status, body weight, physical activity, tobacco use, and nutrition [6, 12–15].

Studies have shown that dietary behavior is an important lifestyle factor impacting on the risk of developing HTN [12, 13]. However, the influences of specific dietary factors on high BP risk for blacks remain uncertain, in part because dietary behaviors and patterns differ over time, across geographical areas, and across demographic subgroups (e.g., north and south, urban and rural, lower and higher socioeconomic status) [16–18].

## Dietary Differences Between Blacks and Whites

The NHANES study (2009–2010) reported that compared with whites, blacks consumed on average lower amounts of whole grains, fruits, and vegetables (0.8, 1.2, 1.3 servings/day for black men, respectively, vs. 1.1, 1.6, 2.1 servings/day for white men) and higher amounts of sugar-sweetened beverages (11.2 servings/day for black men vs. 8.3 servings/day for white men) [5]. Blacks had a higher average intake of dietary cholesterol (311 mg/day for black men vs. 263 mg/day for white men) and lower average intake of dietary fiber compared with that of whites (13.6 g/day for black men vs. 16.3 g/day for white men) [5]. Data from NHANES 2005–2010 showed that blacks consumed a larger percentage of energy from added sugars than that of whites (14.5 % for black men vs. 12.8 % for white men) [19]. NHANES also reported that lower percentages of blacks met the *Dietary Guidelines for Americans* [20] compared with that of whites for whole grains (≥3 servings/day), fruits (≥2 cups/day), vegetables (≥2 cups/day), nuts, legumes and seeds (≥4 servings/week), and sugar-sweetened beverages (≤36 oz./week) [5]. About 11 % of whites and 8 % of blacks met guidelines for fruits; with 100 % fruit juices included, the number of servings increased and the proportions of whites consuming ≥2 cups/day doubled to 26 % and nearly quadrupled in blacks to 29 % [5]. Blacks were 43 % less likely than whites to meet fruit and vegetable guidelines [21]. The 2005 Behavioral Risk Factor Surveillance System (BRFSS) reported that only about 21 % of blacks consumed ≥5 servings/day of fruits and vegetables; the lowest of any US ethnic group [22].

The Reasons for Geographic and Racial Differences in Stroke (REGARDS) Study examined nutrient intakes among 21,334 blacks and whites in the Stroke Belt (non-coastal regions of North Carolina, South Carolina, and Georgia, as well as Alabama, Arkansas, Georgia, Louisiana, Mississippi, and Tennessee; 20 %), Stroke Buckle (coastal plain regions of North Carolina, South Carolina, and Georgia; 30 %), and elsewhere in the USA (50 %) [17, 18]. Compared with whites, blacks within each region consumed a higher percentage of energy from carbohydrates and a lower percentage of energy from fats, and less fiber and alcohol (Table 1) [17, 18]. The daily intakes of Na, K, magnesium (Mg), and calcium (Ca) were lower among black men compared with white men, whereas cholesterol intake was higher in blacks (Table 1) [17]. Black women also had significantly lower intakes of Ca, Mg, K, iron (Fe), and also Na compared to that of white women within each region (Table 1) [18].

**Table 1:** Daily intake of macro/micronutrients of 5,105 men and 7,079 women from the Reasons for Geographic and Racial Differences in Stroke (REGARDS) Study [17, 18]

	Men [17]				Women [18]			
	Stroke belt^a^		Stroke buckle^b^		Stroke belt^a^		Stroke buckle^b^	
	Black (*n* = 793)	White (*n* = 2,456)	Black (*n* = 418)	White (*n* = 1,438)	Black (*n* = 1,600)	White (*n* = 2,603)	Black (*n* = 971)	White (*n* = 1,905)
Carbohydrates, % energy	49.5 (12.6)	47.1 (11.3)**	50.0 (12.1)	46.7 (11.2)**	50.9 (11.9)	48.5 (11.7)**	51.6 (12.3)	48.1 (11.6)**
Protein, % energy	13.6 (3.7)	14.4 (3.6)**	13.7 (3.9)	14.3 (3.9)**	13.7 (4.0)	14.5 (4.1)**	13.8 (4.1)	14.5 (3.8)**
Total fats, % energy	36.2 (9.4)	37.9 (97)**	35.8 (9.7)	37.8 (9.9)**	36.2 (10.1)	37.8 (10.0)**	36.0 (9.5)	37.9 (9.8)**
Alcohol, % energy	0.3 (3.1)	0.4 (4.7)*	0.2 (2.0)	0.5 (5.9)**	0.0 (0.5)	0.1 (1.1)**	0.0 (0.4)	0.2 (1.7)**
Fiber, g	13.5 (10.2)	15.3 (10.3)**	12.6 (9.6)	15.0 (9.6)**	12.8 (9.5)	14.3 (10)**	12.8 (9.6)	13.7 (9.2)**
Cholesterol, mg	222 (200)	215 (159)	239 (216)	207 (159)*	167 (150)	156 (124)*	161 (159)	155 (113)
Sodium, mg	2170 (1476)	2370 (1304)**	2149 (1478)	2321 (1286)**	1854 (1356)	1947 (1186)*	1779 (1352)	1889 (1112)*
Potassium, mg	2306 (1453)	2706 (1393)**	2218 (1379)	2647 (1242)**	2148 (1346)	2464 (1341)**	2093 (1362)	2361 (1276)**
Calcium, mg	559 (405)	666 (455)**	523 (376)	621 (411)**	506 (394)	606 (433)**	460 (394)	572 (419)**
Magnesium, mg	238 (149)	282 (157)**	232 (140)	275 (133)**	218 (138)	255 (147)**	212 (137)	247 (140)**
Iron, mg	11.2 (6.9)	12.8 (7.5)**	11.7 (7.6)	12.7 (7.0)**	10.1 (6.8)	11.0 (6.8)**	9.7 (6.9)	10.5 (6.3)**

^a^Non-coastal regions of North Carolina, South Carolina, and Georgia, as well as Alabama, Arkansas, Georgia, Louisiana, Mississippi, and Tennessee

^b^Coastal plain regions of North Carolina, South Carolina, and Georgia

*P < 0.05 for the Wilcoxon two-sample test within region (black vs. white); **P < 0.001 for the Wilcoxon two-sample test within region (black vs. white)

Data from the Continuing Survey of Food Intakes by Individuals (CSFII) 1994–1998 and NHANES 1999–2000 showed that blacks in all age groups consumed significantly fewer servings/day of total dairy, milk, cheese, and yogurt than that of non-blacks, and blacks in all age groups did not meet dairy recommendations from the *U.S. Dietary Guidelines* (≥3 servings/day) [23]. Black women aged 31–50 in the CSFII had a mean (SD) total dairy intake of 0.71 (0.07) servings/day compared to other women in the same age group of 1.21 (0.03) servings/day. The total dairy intake of black women in the NHANES (0.83 servings/day) was similar to black women reported in the CSFII. Data from NHANES reported that compared with whites (*n* = 8,302), blacks (*n* = 3,458) had lower adjusted mean intakes of total dairy (138.8 g/day for blacks vs. 273.4 g/day for whites), low-fat milk (31.8 vs. 88.9 g/day), yogurt (2.1 vs. 7.1 g/day), and lower intakes of nutrients found in dairy foods (K, Ca, Mg, phosphorus (P), and vitamin D) [24, 25].

Whites were more likely than blacks to use dietary supplements; NHANES 2003–2006 reported that 59 % of whites took dietary supplements compared to 36 % blacks [26, 27]. The Multiethnic Cohort Study investigated multivitamin/mineral intakes of 159,017 participants and reported a higher rate of supplement use in whites (57 %) than blacks (43 %). However, median intakes from supplements for most nutrients (e.g., folate, vitamin B6, vitamin D, Ca) were similar for blacks and whites except for vitamin A, where the median intake was 1,473 μg retinol activity equivalents for whites and 1,233 μg retinol activity equivalents for blacks [28].

Data from NHANES 2007–2010 showed that there was a significant difference between blacks and whites in the percentage of calories consumed from fast foods (defined as foods usually sold at eating establishments for quick availability or takeout [20]) [29]. Young adults aged 20–39 consumed the highest percentage of calories from fast foods (21 % in blacks vs. 15 % in whites, *P* < 0.05). No ethnic differences prevailed in calorie intake from fast foods among adults aged 60 and over.

## Diet and Blood Pressure

**Fig. 1:**
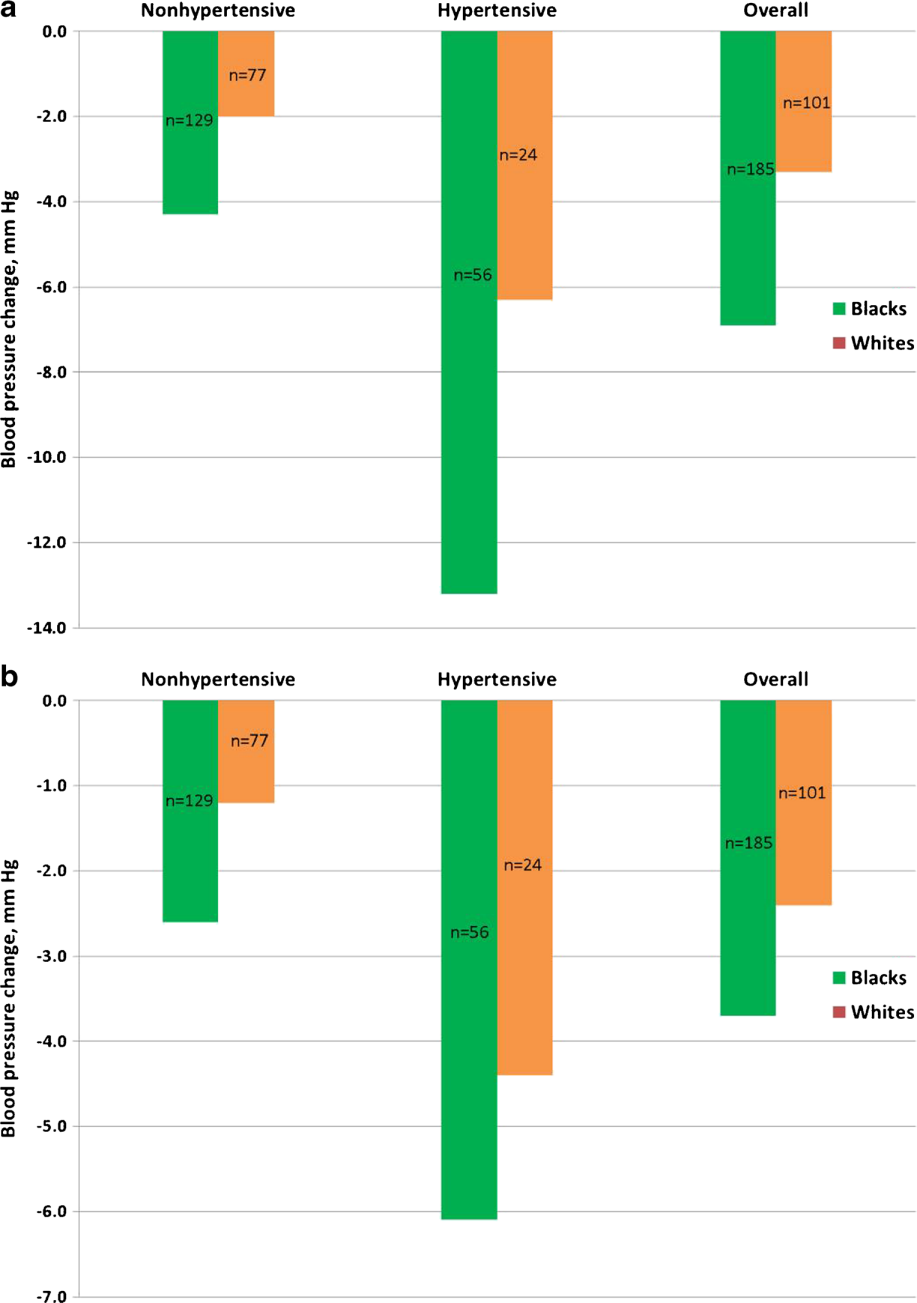
Effect of ethnicity and hypertension status on (**a**) systolic blood pressure and (**b**) diastolic blood pressure response to Dietary Approaches to Stop Hypertension (DASH) combination diet, adjusted for site and cohort effect [31]

The Dietary Approaches to Stop Hypertension (DASH) and the DASH-Na trials demonstrated that dietary patterns rich in vegetables, fruits, and low-fat dairy products and reduced in Na, total fat, saturated fat, and dietary cholesterol lower BP effectively in prehypertensive and hypertensive adults, blacks and whites [30–35]. In the DASH trial, 459 participants (SBP <160 mmHg and DBP 80–95 mmHg) were randomly assigned to a control diet (low in fruits, vegetables, and dairy products, with a fat content typical of the average diet in USA), fruits-and-vegetables diet (rich in fruits and vegetables), or combination diet (rich in fruits, vegetables, and low-fat dairy products and with reduced saturated and total fats) [30]. With the combination diet, SBP was lower by 5.5 mmHg (95 % confidence interval (CI), 3.7–7.4 mmHg) than that with the control diet (*P* < 0.001). With the fruits-and-vegetables diet, SBP was lower by 2.8 mmHg (0.9–4.7 mmHg) than that with the control diet (*P* < 0.001). Compared with the fruits-and-vegetables diet, the combination diet reduced SBP by 2.7 mmHg (0.9–4.6) more (*P* < 0.001) [30]. The DASH combination diet lowered BP significantly more in blacks than in whites (Fig. 1) despite similar BP levels at baseline (131.8/84.8 mmHg for blacks vs. 130.9/84.5 mmHg for whites) [31].

In the DASH-Na trial, 412 participants (with SBP 120–159 mmHg and DBP 80–95 mmHg) were randomly assigned to a control diet or the DASH diet; within the assigned diet, participants ate foods with three different levels of Na (for a 2100-kcal diet: lower 50 mmol/day, intermediate 100 mmol/day, and higher 150 mmol/day) [32]. The reduction of Na intake from the high to the intermediate level lowered SBP by 2.1 mmHg (95 % CI 0.8–3.4 mmHg, *P* < 0.001) with the control diet and by 1.3 mmHg (95 % CI 0.0–2.6 mmHg, *P* = 0.03) with the DASH diet. The reduction of Na intake from the intermediate to the low level resulted in additional BP lowering of 4.6 mmHg (95 % CI 3.2–5.9 mmHg) with the control diet (*P* < 0.001) and 1.7 mmHg (95 % CI 0.4–3.0 mmHg) with the DASH diet (*P* < 0.01) [34]. The baseline BP levels were similar for blacks and whites; average SBP/DBP was 135.3/86.1 mmHg for blacks and 134.1/85.1 mmHg for whites. Among participants on the control diet, lower (vs. higher) Na intake decreased SBP by 8.0 (95 % CI 6.5–9.4 mmHg) in blacks and by 5.1 mmHg (3.4–6.7) in whites (*P* < 0.01). Among participants on the DASH diet, lower (vs. higher) Na intake decreased SBP by 3.6 mmHg (95 % CI 2.2–5.1) in blacks and by 2.2 mmHg (0.5–3.8) in whites [36, 37].

The DASH/DASH-Na diet BP reduction was more pronounced for blacks compared to whites [31, 36, 37]. Although the DASH dietary approach has been incorporated into lifestyle changes recommended for patients with HTN [3], data show that few hypertensive Americans consume diets even modestly concordant with the DASH diet and less so for blacks [38]. Only about 19 % of individuals with known HTN from NHANES 1999–2004 had DASH-concordant diets.

The Exercise and Nutrition Interventions for Cardiovascular Health Study (ENCORE), a 16-week intervention trial of 144 participants with high BP (SBP 130–159 mmHg and/or DBP 85–99 mmHg), reported that greater adherence to the DASH diet was associated with larger BP reductions [39•]. Each 2-point increase in DASH diet adherence was associated with a 3.4 mmHg (95 % CI 2.4 to 4.4) reduction in SBP. The DASH adherence score (adopted from Folsom and colleagues) [40], ranging from 0 to 10, was calculated from a food frequency questionnaire (FFQ). At baseline, the DASH adherence score was 3.40 in blacks and 3.91 in whites. Black participants in the trial were less likely to consume foods consistent with the DASH diet compared with that of whites; they consumed fewer low-fat dairy products and more sweets compared with that of whites. After intervention, participants increased their consumption of DASH-designated foods, and the DASH adherence score increased to 4.68 in blacks and 5.83 in whites (*P* < 0.001); compared with whites, blacks continued to consume more meats, sweets, fats, and fewer fruits. These findings indicated lower adherence by black than white participants to the DASH diet and, in turn, smaller BP reduction independent of weight loss.

The Atherosclerosis Risk in Communities (ARIC) study analyzed FFQ data for 8,208 non-hypertensive women and men aged 45 to 64 years. Whites consuming ≥3 daily servings of low-fat milk, compared with those consuming <1 serving, had a 2.7 mmHg smaller SBP increase with a 9-year follow-up (*P* for trend = 0.01) [41]. Dairy product intake was not associated with changes in BP in blacks. The ARIC Study also reported that P intake was inversely associated with SBP [42]. Compared with participants in the lowest quintile of P intake at baseline, those in the highest quintile had significantly lower baseline SBP for both whites and blacks after adjustment for non-dietary confounders (−2.3 mmHg, 95 % CI −3.4 to −1.2, *P* for trend <0.0001 for whites; −2.3 mmHg, −5.5 to 0.8, *P* for trend = 0.01 for blacks), but not after additional adjustment for dietary confounders (−2.9 mmHg, 95 % CI −4.7 to −1.1, *P* for trend = 0.002 for whites; −0.8 mmHg, −6.5 to 4.9, *P* for trend = 0.63 for blacks). Further analyses showed that P from dairy products (the main source of P with 31 % contribution)—but not from other sources (fish for 7 % and red meat for 7 % of P)—was associated with lower baseline BP and reduced risk of HTN.

NHANES 1999–2004 reported that BP was inversely and significantly (*P* < 0.05) associated with fluid milk, yogurt, and Mg [25], while cheese was positively and significantly associated with SBP and DBP. The adjusted mean SBP was 125.7 mmHg for blacks and 122.6 mmHg for whites (*P* < 0.05); this SBP difference between blacks and whites was partly explained by dairy-related nutrients.

The International Study of Macro/Micronutrients and Blood Pressure (INTERMAP) of 2,195 men and women aged 40 to 59 from 8 US population samples reported that less favorable intakes of multiple foods/nutrients by blacks than whites partly accounted for the higher BP of blacks [43•]. The average BP was 124.2/78.4 mmHg for black men and 123.9/75.1 mmHg for black women and 120.0/75.9 mmHg for white men and 114.5/70.6 mmHg for white women. Of black women, 47 % were hypertensive compared to 20 % of white women (*P* < 0.0001). Compared with whites, blacks had lower average intake of fresh fruits, total vegetables, total grains, bread/rolls, and cheese, and higher intake of processed meats, pork, eggs, fruit juice/drinks, sugar-sweetened drinks, fish, and poultry (Table 2). Black participants also had lower average intakes of vegetable protein, glutamic acid, starch, fiber, Ca, Mg, P, and Fe, and lower urinary K excretion, along with higher intakes of dietary cholesterol, total sugars, fructose/glucose/sucrose, glycine and higher urinary Na/K ratio, related to higher black BP (Table 2). Compared with whites, mean SBP of blacks was higher by 4.8 mmHg for men (*P* < 0.001) and 9.0 mmHg for women adjusted for non-dietary confounders (*P* < 0.0001). With additional adjustment for nutrients, the effects on black-white SBP difference reduced to 2.3 mmHg (52 % reduction) in men and 5.3 mmHg (21 % reduction) in women. The additional combinations of foods and urinary metabolites had little further influence on higher BP in blacks.

**Table 2:** Average intake of foods, macro/micronutrients, of 785 men and 774 women from the International Study of Macro/Micronutrients and Blood Pressure (INTERMAP) [43•]

	Men		Women	
	Black (*n* = 165)	White (*n* = 620)	Black (*n* = 204)	White (*n* = 570)
Fresh fruit, g/1,000 kcal	42.1 (54.0)	53.0 (64.8)*	57.4 (73.5)	73.2 (67.9)**
Total vegetable, g/1,000 kcal	107.2 (60.2)	127.2 (65.6)***	133.9 (79.6)	144.5 (74.9)
Total grains, g/1,000 kcal	89.0 (40.4)	97.3 (36.4)*	85.0 (33.8)	106.2 (37.0)***
Bread/rolls/biscuits, g/1,000 kcal	30.7 (16.3)	38.2 (21.0)***	31.3 (19.7)	39.4 (22.6)***
Cheese, g/1,000 kcal	6.8 (6.4)	13.1 (12.1)***	9.2 (9.3)	15.6 (14.8)***
Processed meats, g/1,000 kcal	11.1 (12.0)	9.9 (11.9)	10.3 (13.5)	6.9 (9.4)***
Pork, g/1,000 kcal	10.0 (13.2)	8.5 (11.8)	10.0 (15.5)	6.3 (9.9)***
Eggs, g/1,000 kcal	12.5 (13.1)	9.9 (9.9)**	12.4 (12.7)	9.4 (9.1)***
Fruit juices/drinks, g/1,000 kcal	122.5 (122.7)	69.9 (102.1)***	110.1 (126.0)	66.4 (118.6)***
Sugar-sweetened beverages, g/1,000 kcal	210.1 (165.6)	126.3 (150.9)***	194.7 (166.7)	76.7 (108.0)***
Fish/fish roe/shellfish, g/1,000 kcal	10.2 (16.3)	7.5 (12.8)*	9.9 (14.6)	8.5 (13.7)
Poultry, g/1,000 kcal	27.6 (22.6)	17.1 (16.8)***	30.1 (23.7)	17.8 (17.0)***
Vegetable protein, %kcal	4.4 (1.7)	5.0 (1.5)***	4.5 (1.3)	5.4 (1.4)***
Glutamic acid, %kcal	2.8 (0.5)	3.0 (0.6)***	2.8 (0.6)	3.1 (0.5)***
Glycine, %kcal	0.7 (0.2)	0.6 (0.2)***	0.7 (0.2)	0.6 (0.2)***
Starch, %kcal	19.9 (5.4)	21.9 (5.0)***	19.8 (5.0)	23.2 (5.0)***
Total sugars, %kcal	28.6 (8.7)	26.6 (8.4)***	29.8 (8.5)	27.7 (7.2)***
Fructose/glucose/sucrose, %kcal	24.2 (8.8)	20.7 (8.4)***	25.1 (8.6)	20.8 (6.9)***
Total dietary fiber, g/1,000 kcal	7.6 (3.4)	8.9 (3.3)***	8.2 (3.4)	9.9 (3.4)***
Dietary cholesterol, mg/1,000 kcal	140.7 (62.1)	123.6 (52.9)***	141.9 (64.1)	117.4 (48.6)***
Urinary potassium, mmol/24 h	55.5 (17.9)	71.7 (21.2)***	44.2 (15.9)	56.8 (17.9)***
Urinary sodium/potassium ratio	3.55 (1.28)	2.75 (0.98)***	3.69 (1.53)	2.68 (0.98)***
Calcium, mg/1,000 kcal	288.9 (107.3)	382.3 (138.4)***	308.8 (114.4)	430.1 (147.4)***
Magnesium, mg/1,000 kcal	125.4 (37.0)	147.6 (35.1)***	130.4 (37.6)	159.7 (39.0)***
Iron, mg/1,000 kcal	6.9 (3.1)	7.8 (2.9)***	7.0 (2.3)	8.3 (2.8)***
Non-heme iron, mg/1,000 kcal	6.3 (3.1)	7.3 (2.9)***	6.5 (2.2)	7.8 (2.8)***
Phosphorus, mg/1,000 kcal	523.9 (103.3)	600.9 (125.7)***	534.4 (117.5)	629.9 (127.9)***

*P < 0.05 for the Student’s *t* test; **P < 0.01 for the Student’s *t* test; ***P < 0.001 for Student’s the *t* test

The Heart Follow-Up Study (HFUS), an investigation of 1,568 men and women in New York City, using 24-h urine collection to assess Na and K intake, found that black participants had higher weighted mean SBP (126.0 mmHg for blacks vs. 121.3 mmHg for whites, *P* < 0.01) and DBP (77.2 vs. 72.8 mmHg, *P* < 0.001) compared with that of whites [44]. Black men had significantly higher Na and Na/K ratio and lower K intakes than that of white men (*P* < 0.001); black women had higher Na/K ratio and lower K intake compared with that of white women (*P* < 0.001). Among black men aged ≤50, 1,000 mg greater Na intake was associated with 1.9 mmHg higher in SBP (*P* < 0.05); 1,000 mg higher K intake was associated with 4.1 mmHg lower in SBP (*P* < 0.05); 1 unit higher in Na/K ratio was associated with 3.4 mmHg higher in SBP (*P* < 0.01). No significant relationships between Na, K intakes, and SBP were found in white men of the same age group.

The Jackson Heart Study (JHS), using a validated FFQ to assess nutrition among 1,775 blacks in the southern USA (Jackson, MS), identified three major dietary patterns: “southern,” “fast food,” and “prudent,” and reported that higher adjusted southern pattern scores were significantly associated with higher odds ratio (OR) for HTN (*P* < 0.05) [45]. The southern dietary pattern was characterized by the high consumption of vegetables with high starch content (e.g., beans, corn, and potatoes), fried meats, poultry, and fish, margarine and butter for cooking, and whole milk and buttermilk for cornbread and rolls. The fast food pattern was characterized by high consumption of sugar-sweetened drinks, salty snacks, and fast foods, and the prudent pattern was characterized by high intakes of fruits and vegetables and cereals and low intakes of white bread and sweets. After adjustment for non-dietary confounders, higher southern pattern scores (OR = 1.42, 95 % CI 1.1–1.9 for tertile 2, *P* = 0.02 and OR = 1.14, 95 % CI 0.7–1.8 for tertile 3, *P* = 0.6) and higher fast food pattern scores (OR = 1.35, 95 % CI 0.9–1.8 for tertile 2, *P* = 0.06 and OR = 1.67, 95 % CI 1.1–2.7 for tertile 3, *P* = 0.03) were significantly associated with HTN. The prudent pattern score was inversely associated with HTN (OR = 0.75, 95 % CI 0.6–0.9 in tertile 2, *P* = 0.05 and OR = 0.69, 0.5–0.9 in tertile 3, *P* = 0.02).

## Conclusions

Blood pressure levels have consistently been found to be higher in blacks than whites, with an earlier onset of HTN. Data showed blacks had significantly lower intakes of fruits, vegetables, and dairy products, and lower intakes of K, Mg, Ca, and P compared with that of whites. However, dietary differences between blacks and whites, including Na and K intakes, do not fully explain the higher prevalence of adverse BP levels in blacks. The DASH diet with Na reduction resulted in a greater reduction of BP in blacks than whites. Lower average incomes for blacks compared to whites and strong cultural influences relating to food preferences, food preparation, and perceptions about eating practices may make it more challenging for blacks to adhere to a DASH-type diet. African-Americans have a distinctive culinary heritage with diverse flavors (derived from the African continent, the West Indies, and North America). Ethnic variations in food choices are poorly reflected in the DASH diet. More work is needed on the implementation of the DASH BP reduction diet for blacks, including ways to identify affordable nutrient-rich foods and reduce consumption of fried, energy-dense, salt-dense, and nutrient-poor foods [46].
